# A Weighted-Transfer Domain-Adaptation Network Applied to Unmanned Aerial Vehicle Fault Diagnosis

**DOI:** 10.3390/s25061924

**Published:** 2025-03-19

**Authors:** Jian Yang, Hairong Chu, Lihong Guo, Xinhong Ge

**Affiliations:** 1 Changchun Institute of Optics, Fine Mechanics and Physics, Chinese Academy of Sciences, Changchun 130033, China; ncortexyj@126.com (J.Y.); guolh@ciomp.ac.cn (L.G.); gexin820209@126.com (X.G.); 2 School of Optoelectronics, University of Chinese Academy of Sciences, Beijing 100049, China

**Keywords:** intelligent fault diagnosis, UAV, transfer learning, domain adaptation

## Abstract

With the development of UAV technology, the composition of UAVs has become increasingly complex, interconnected, and tightly coupled. Fault features are characterized by weakness, nonlinearity, coupling, and uncertainty. A promising approach is the use of deep learning methods, which can effectively extract useful diagnostic information from weak, coupled, nonlinear data from inputs with background noise. However, due to the diversity of flight environments and missions, the distribution of the obtained sample data varies. The types of fault data and corresponding labels under different conditions are unknown, and it is time-consuming and expensive to label sample data. These challenges reduce the performance of traditional deep learning models in anomaly detection. To overcome these challenges, a novel weighted-transfer domain-adaptation network (WTDAN) method is introduced to realize the online anomaly detection and fault diagnosis of UAV electromagnetic-sensitive flight data. The method is based on unsupervised transfer learning, which can transfer the knowledge learnt from existing datasets to solve problems in the target domain. The method contains three novel multiscale modules: a feature extractor, used to extract multidimensional features from the input; a domain discriminator, used to improve the imbalance of the data distribution between the source domain and the target domain; and a label classifier, used to classify data categories for the target domain. Multilayer domain adaptation is used to reduce the distance between the source domain datasets and the target domain datasets distributions. The WTDAN assigns different weights to the source domain samples in order to weight the different contributions of source samples to solve the problem during the training process. The dataset adopts not only open datasets from the website but also test datasets from experiments to evaluate the transferability of the proposed WTDAN model. The experimental results show that, under the condition of fewer anomalous target data samples, the proposed method had a classification accuracy of up to 90%, which is higher than that of the other compared methods, and performed with superior transferability on the cross-domain datasets. The capability of fault diagnosis can provide a novel method for online anomaly detection and the prognostics and health management (PHM) of UAVs, which, in turn, would improve the reliability, repairability, and safety of UAV systems.

## 1. Introduction

With the rapid development of semiconductor electronics, computers, the internet, communications, and other technologies, new devices are becoming more intelligent. As the density of electronic modules is growing geometrically on new devices, the safety and reliability of new devices are facing increasing challenges.

UAVs, as one of the representative products in these types of new modern equipment, have a wide range of applications in various fields due to their fast mobility, high execution efficiency, low cost, and ease of use. In recent years, the intelligence level in UAVs has improved rapidly. Their airborne platforms are heavily equipped with electronic devices, which exhibit high transmitting power and high density and operate with high sensitivity. However, UAVs are limited by their size, dense equipment installation, and limited space isolation, and these electronic devices are susceptible to internal and external electromagnetic signal interferences, which make them prone to electromagnetic compatibility problems. When UAVs encounter extreme weather or high-power electromagnetic interference weapons, a high transient electromagnetic pulse can couple to the airborne receiving devices, causing interference at a distance and implementing destruction in the near distance. The communication, control, and other electronic systems on the UAV will encounter harsh electromagnetic environments that could ultimately result in a wide variety of fault problems, seriously affecting the work efficiency and life expectancy of UAVs.

A UAV is operated without any pilots in the execution of missions. When faults occur, emergency measures cannot be taken in a timely manner, so there is a large difference in terms of the reliability and safety between UAVs and manned aircraft. According to a report by the *Washington Post*, the catastrophic accident rate for drone flights is one to two orders of magnitude higher than that for manned aircraft. Descriptions of recent reports of related UAV accidents [1] indicate that, when an accident occurs, it is usually characterized by complexity and no obvious regularity; fault characteristics present at multiple levels with interconnectivity, presenting uncertainty with regard to the time of the occurrence of the fault. It is now more difficult to identify the fault problem and to carry out a correct analysis than before. These issues lead to increasingly complex challenges for the safety and reliability of UAVs. According to recent statistics, the U.S. military has crashed nearly 500 UAVs worldwide due to sensor, software, and key component faults, resulting in direct economic losses of nearly USD 1 billion [2]. The maintenance cost of a UAV from manufacturing to decommissioning in developed countries accounts for more than 70% of the total cost of the UAV lifespan [3]. UAV flight data can reflect the working status information of specific equipment, and they are the main means for assessing whether a UAV is in an abnormal state. Catastrophic loss of UAVs can be avoided if anomaly data are detected before or during the pre-malfunction stage. As a wide variety of sensors and measurement modules are integrated into UAVs, these sensors are used to measure the data on UAVs and their surroundings to ensure the safety and stability of UAVs during their missions. The sensors are characterized by high sensitivity and a wide frequency spectrum, making them sensitive sources that are highly sensitive to electromagnetic interference [4]. In some harsh electromagnetic environments, the data collected by the sensors are prone to large deviations. These deviation signals can cause UAV control systems to send the wrong commands, which, in turn, lead to flight safety hazards, or even failures and crashes. Therefore, the occurrence of faults in system components can be minimized or avoided if the anomaly data can be detected before or during the pre-malfunction stage by analyzing the sensor data. 

There have been many intelligent fault-diagnosis methods that take UAV sensors as research objects; these mainly include analytical-model-based, knowledge-based, and data-driven methods. The analytical-model-based method identifies faults by constructing accurate physical models to characterize the UAV and by comparing the output of the constructed model to the real output. Kang Xu et al. [5] calculated the cosine value of an attribute and the parallel line in the direction of that attribute, screened the anomalous related attributes by the metric cosine value for telemetry data with too-high dimensionality and then used the screened attributes for anomaly detection. Skormin V et al. [6] proposed a method based on recursive least squares (RLS), which utilizes RLS to track and estimate flight-control-related parameters in real time; then, it detects instantaneous anomaly flight data by measuring the difference between the true and estimated values of the parameters tracked by the flight data. However, it is difficult to construct a UAV system model because the composition of the UAV system is fine and complex and the fault diagnosis performance is sensitive to modeling errors, parameter noise, and external interference factors, so the robustness of the method is poor in practical applications. 

The knowledge-based approach constructs models using cause-and-effect modeling and expert prior knowledge from qualitative descriptions; these include expert systems, fault tree reasoning, and the fuzzy reasoning method. Xuechu Sun [7] established a knowledge system for UAVs based on the structural diagram of fault causes; using positive and negative integrated reasoning and subjective Bayesian reasoning, they improved the fault recognition accuracy of the expert system. Hassl designed and applied a fault tree analysis software in Boeing’s aircraft health management system [8]. Constructing a model requires a comprehensive understanding and accurate representation of the system, but fault knowledge of UAVs is difficult to access comprehensively; so, it is hard to build a large and comprehensive expert knowledge base model. Accordingly, our ability to discriminate among UAV fault diagnoses is limited.

In recent years, with the rapid development and application of computers and artificial intelligence technologies, data-driven methods that use deep learning have become a popular research direction among experts. Using deep neural network models to learn fault features from UAV flight data and then identify faults is an effective approach in improving the efficiency of UAV fault diagnosis approaches and enhancing the available level of predictive maintenance. Jiaojiao Hu proposes an anomaly-detection method based on a convolution neural network (CNN) model. Considering the imbalance of samples in each category, the method first used a sampling method to process the time–series data in each category; then, it used the processed data to train the CNN model in a supervised way; finally, it was able to distinguish between normal and anomaly sequence samples through time–series classification [9]. Janakiraman V M et al. [10] utilized an automatic discovery of precursors in time–series (ADOPT) approach based on reinforcement learning; this transforms the anomaly-detection problem into a suboptimal decision-modeling problem. The method achieved good performances in aircraft stall detection. Hanze Liu proposed an intelligent diagnosis method by using a cavity convolutional neural network that was built based on knowledge of flight control fault diagnosis. An intelligent fault-identification system software was designed to adapt to the flight-control system of a certain model of UAV; this can intelligently evaluate the flight control state of a UAV, realizing the effective diagnosis of UAV flight control faults [11]. Gao et al. [12] implemented fault diagnosis in inertial sensors, utilizing the Hilbert–Yellow transform approach and a CNN model based on bi-directional long- and short-term memory networks. Wei Li proposed the stacked denoising autoencoder (SDA) fault-diagnosis method, which solved the problem of insufficient network generalization performance by training noisy data as samples; finally, the approach was able to accurately identify the type of actuator fault [13].

Data-driven methods with high fault recognition capabilities rely on two conditions: the same or similar data sample distribution and complete data labeling. However, due to the complex composition of the UAV system and the missions in different application scenarios, the sample distributions are various. It is difficult for a model trained using a specific data distribution to perform well on another dataset with a different distribution. On the other hand, generally, UAVs work in safe flight conditions, so the probability of failure is small during specific monitoring periods. So, the acquisition of fault sample data and labels is time-consuming and the data are scarce. Insufficient fault sample data and labels limit the training dimension of the model, resulting in the poor generalization performance of a given model. Several scholars have proposed some novel methods for improving the performance of models under small-sample conditions. Dong Y et al. [14] devised a multiscale adaptive feature extractor (MAFE) to effectively mine signal features and utilized a compound attention mechanism to reweight the features that were extracted from the MAFE. He also constructed a dynamic normalized supervised contrastive loss function; the loss function can adjust the distributional distance between different categories to the optimal degree by balancing the contributions among different sample sets. But the method cannot recognize unknown classes of faults, which limits its further application in the industry. Liu Y et al. [15] proposed a few-shot contrastive learning method for intelligent fault diagnosis with limited samples. Discriminative representations are exploited with counterfactual augmentation, which considers the compatibility between data distributions and feature mappings as well as the balance between global associations and local diffusions. However, it is still tough to diagnose faults with this method in real time. The disadvantages of these methods limit the wide application of deep learning methods for intelligent fault diagnosis under small sample sets.

Transfer learning is a branch of deep learning; it can transfer existing knowledge and experience from one domain to solve problems in different but similar or related domains [16]. It saves the cost of re-collecting large numbers of labeled samples when the target task is lacking in high-quality training data. Some scholars have introduced the transfer learning method into fault diagnosis for equipment to overcome the scarcity of labeled samples in their models. Grubinger et al. [17] used the transfer component analysis (TCA) method, which is based on the use of maximum mean discrepancy (MMD), as the distance metric function between two domains; this is then added to the final loss function to align the distributions of the source and target domains. Thus, it extracts the features that are shared between the two domains. Wenyuan Dai [16] proposed a method called TrAdaBoost based on the Adaboosting method, in which the algorithm iterates to increase the weights of the samples that are beneficial to the target task and decreases the weights of the samples that are detrimental to the target task. These weighted source data will be used as auxiliary training data, which will be trained together with the target domain data to improve the learning effect of the target domain data. The method is more applicable in dealing with symmetric binary classification problems and cannot be adapted to application scenarios where the proportions of positive and negative samples are severely unbalanced. The distribution difference between the source and target domain can be reduced through introducing the domain-adaptation method. Some scholars have also introduced generative adversarial networks into transfer learning to scale down discrete sample distribution. Generative adversarial networks (GANs) [18] can generate high-quality pseudo-samples to improve the class imbalance among samples. Xin Wang [19] proposed a trackable generative adversarial network which can achieve more comprehensive features from different domains. The model is robust for fault diagnosis in rotating machinery. Ganin Y et al. [20] first introduced the adversarial idea into transfer learning and proposed the DANN model. In this model, the feature extraction network replaces the generative network, extracting the features of the samples; here, the role of the discriminator is to identify whether the extracted features belong to the source domain or the target domain after the feature extraction network has been applied. Tzeng E et al. [21] summarized the characteristics of generative networks and transfer learning; they proposed a general framework for transfer learning based on the adversarial idea. When the adversarial training between the feature extraction network and the discriminative network reaches the Nash equilibrium, the feature extraction network extracts the features that are indistinguishable from the discriminative network, namely the transfer of the shared features; here, the classifiers trained on the basis of the idea are used to directly classify the target domain data.

In summary, the transfer learning method can reuse existing sufficient knowledge of fault data to solve the target domain problem; this process is insensitive to the scarcity of available training samples, and it can overcome the limitations of the traditional deep learning methods for UAV fault diagnosis that were mentioned above. We proposed a weighted-transfer domain-adaptation network (WTDAN) based on unsupervised learning, taking the typical faults of sensors in UAV flight-control system as the research object. This approach is a combination of the transfer learning method and the domain adversarial adaptation method. Here, the transfer learning method part can transfer the knowledge obtained from the existing data to the target domain; the domain adversarial adaptation part can improve the imbalance of the data distribution between different domains through mutual adversarial training [22], and it reduces the distribution discrepancy between datasets. The method assigns different weights to the source domain samples in order to weight the different contribution of source samples to the model during training. Accordingly, it can establish a better fault-diagnosis model for online anomaly detection and fault diagnosis. The innovations and contributions of this paper are summarized as follows: 

(1) The proposed model constructs an unsupervised transfer learning method by combining three neural network modules. It utilizes the fault knowledge learned from labeled sensor fault datasets through training to perform online anomaly monitoring and fault identification on the acquired UAV sensor data.

(2) The feature extractor not only undertakes the extraction of high-dimensional features from the input data but also works together with the domain discriminator to generate high-quality pseudo target domain data in adversarial training to improve the imbalance of samples between different domains. The domain discriminator accumulates common features across domains in the process of discriminating the domains to which the samples belong. The multilayer domain-adaptation loss adapts to transfer training to enhance the capability of domain confusion.

(3) The model uses the loss function to measure the predictive performance of the model quantitatively and improves the performance by optimizing the loss function parameters. In order to weight the different contributions of the source samples during the training process, the model assigns different weights to the source domain samples.

The rest of this paper is organized as follows. The problem definition and basic theory are introduced in Section 2. Then, the proposed method is presented in Section 3, including the framework of the model and the optimization strategy. In Section 4, the performance of the method is validated using different tests and this is compared with the performances of other methods. Some prospects for future work are discussed in Section 5. Finally, the conclusion is summarized in Section 6. 

## 2. Preliminaries

### 2.1. Problem Definition

The source domain data and target domain data are obtained from different sensors and different conditions. Let S and T denote the source domain and the target domain, respectively. $X^{S}=\{(x_{i}^{S},y_{i}^{S}){\}}_{i=1}^{n_{S}}$ denotes the source domain dataset with $n_{S}$ label samples, where $x_{i}^{S}$ represents the ith sample of the source domain dataset, and $y_{i}^{S}$ represents the corresponding label. Similarly, $X^{T}=\{(x_{j}^{T}){\}}_{j=1}^{n_{T}}$ denotes the target domain dataset with $n_{T}$ unlabeled samples, where $x_{j}^{T}$ represents the jth sample of the target domain dataset without label information. $X^{S}$ and $X^{T}$ are subject to the marginal probability distributions, p  and q, respectively, and p≠q. 

### 2.2. Convolutional Neural Network (CNN)

A convolution neural network is a type of feedforward neural network that contains convolutional computation and has a deep structure [23]. CNNs have the capability to conduct feature representation learning, and they can achieve translation-invariant classification from the hierarchical structure of input information. CNNs follow the structure of muti-layer perceptual machines; they generally consist of an input layer, a convolution layer, a pooling layer, fully connected layers, and an output layer. Each node in the input layer corresponds to the original input data, and the dimensions of the input data correspond to the dimensions of the convolutional network. The convolution layer converts the input data to a multidimensional space through nonlinear mapping and extracts the features from the original input. The convolution operation can be expressed as follows:

(1) $$x_{c}^{l}=f^{l}\left(\sum_{i=1}^{C^{l-1}}W_{i,c}^{l}\;*\;x_{i,c}^{l-1}+b_{c}^{l}\right)$$

where $x_{c}^{l}$ denotes the Cth channel output in the layer l; $x_{i}^{l-1}$ denotes the Cth channel the ith input in the layer l−1, which has a convolution kernel cluster $W_{i,c}^{l}$, i = 1, 2…$C^{l-1}$, where $C^{l-1}$ is the number of channels in layer l−1; ∗ denotes the convolution operation; $b_{c}^{l}$ denotes the bias; $f^{l}\left(\cdot \right)$ denotes the activation function, e.g., ReLU.

The pooling layer is placed between successive convolutional layers; this can prevent overfitting and reduce the redundancy of unnecessary feature information and parameters. The pooling method mainly includes maximum pooling and average pooling, as follows:

(2) $$o^{l}\left(i\right)=\underset{\left(i-1\right)W+1\leq r\leq W}{max}o^{l-1}(t)$$

(3) $$o^{l}\left(i\right)=\underset{\left(i-1\right)W+1\leq r\leq W}{avg}o^{l-1}(t)$$

$o^{l}$(i) is the output of the pooling in the layer l, $o^{l-1}\left(t\right)$ is the output of the l−1th convolution layer. W is the width of the local region, i.e., the kernel size of the pooling.

The fully connected layer is located at the tail of CNN; usually, a Softmax regression model is used here to achieve multiple classifications. The Softmax function turns arbitrary real values into probabilities; it is expressed as follows:

(4) $$Q^{j}\left(z\right)=\frac{e^{z^{j}}}{\sum_{k=1}^{K}e^{z^{k}}}$$

where $z^{j}$ is the jth input feature of the activation function; K is the number of categories.

The output layer outputs the final results, setting the number of network nodes according to the number of categories.

### 2.3. Generative Adversarial Network (GAN) 

Generative adversarial networks are inspired by two-player games in game theory. GNNs consist of a pair of generative networks and a discriminative network, where the generative network is used to generate pseudo-real samples from the input noise. This is called the generator. The discriminative network is used to discriminate the authenticity of the samples, and is called the discriminator. The core idea of GANs is to train a generator and a discriminator against each other by repeating a process over and over again, so that they can reach a state of dynamic equilibrium. Finally, the network can achieve high-quality pseudo samples that are similar to real samples. The objective function of GANs is defined as follows:

(5) $$\begin{array}{c} \underset{G}{min}\underset{D}{max}E_{{x\sim P}_{data}}[\log\left(D(x)\right)+E_{{z\sim P}_{noise}}\log(1-D\left(G\left(z\right)\right))] \end{array}$$

where D is the discriminator, G  is the generator, x is the real existed sample, and z is the input noise. $P_{data}$ is the real sample distribution, while $P_{noise}\;$ is the noise distribution. D requires that the loss is as large as possible to maximize the discrimination of data authenticity. G requires that the loss be as small as possible to make the samples generated by the generator more realistic. G and D are alternately updated in a mutual adversarial game until the generator is able to generate data that meet the requirements of the task.

## 3. The Proposed Method

The sample distributions of the obtained datasets are different in various types of missions. And it is time-consuming and expensive to obtain fault samples and their corresponding labels. There is rich knowledge available in open datasets online, which can be combined with the monitoring data that were obtained under laboratory conditions. The proposed objective of the method is to transform these data, allowing us to solve the problem in the target domain. So, there are two missions in this model: one mission is to transfer knowledge from the existing source domain to the target domain data; the other mission is to identify the fault data in the target domain.

### 3.1. Framework of the WTDAN Model 

**Figure 1:**
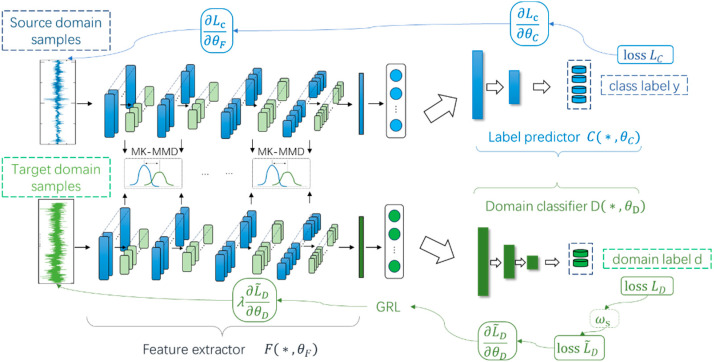
The framework of WTDAN.

The method consists of three components: the first part contains three multiscale modules, i.e., the feature extractor (F), the domain discriminator (D), and the label classifier (C). The feature extractor is used to map the input to a specific space then obtain multidimensional features; the domain discriminator is used to confuse the source domain data with the target domain data, and it cannot distinguish whether the data comes from the source or the target domain, nor can is extract common features across domains, namely the domain-invariant features; the label classifier is used to identify data features from the output of the feature extractor and classify their categories. The core of this part is the interaction of the adversarial training between the domain discriminator and the feature extractor. The domain discriminator is regarded as the discriminator in the generative adversarial network, and the feature extractor is regarded as the generator; however, the input noise is exchanged for the target domain data in this method, and the imbalance of the data distribution between different domains is gradually reduced through continuous adversarial training. The model places a gradient reversal layer between F and D; the function is to reverse the gradient direction in the training process of backpropagation and achieve constant transmission in forward-propagation. The trained method is directly applied in order to classify the target domain data. The framework is shown in Figure 1.

### 3.2. Multilayer Domain Adaptation

The discrepancy between the source domain and the target domain distributions determine the performance of the model. In order to quantitatively measure and reduce the distance between the source domain dataset and the target domain dataset, we used multi-kernel maximum mean discrepancy (MK-MMD) to minimize the differences among the different layers. MMD is a nonparametric distance metric that can be used to measure distribution discrepancies between two datasets [24]. This approach maps datasets to the reproducing Kemel Hilbert space (RKHS) using the differences among the means in the RKHS as a measure of the distance between the two domain data distributions [25]. The MMD between $X^{S}$ and $X^{T}$ is defined as follows:

(6) $$MMD^{2}\left(X^{S},X^{T}\right)={∥\frac{1}{n^{s}}\sum_{i=1}^{n_{s}}\phi \left(x_{j}^{S}\right)-\frac{1}{n^{t}}\sum_{j=1}^{n_{T}}\phi \left(x_{j}^{T}\right)∥}_{H}$$

where H represents the RKHS; $\phi \left(\cdot \right)$ is the nonlinear mapping function from the original feature space to the RKHS. By minimizing the MMD, the desired nonlinear mapping solution can be found. The multicore approach can use different cores to enhance the effect of MK-MMD, so MK-MMD can achieve an optimal kernel option through the linear combination of multiple kernels [26]. MK-MMD has specific k RKHSs compared to MMD. $k\left(x_{i}^{S},x_{j}^{T}\right)$ is defined as a convex combination of m PSD kernels. It converts the traditional problem of kernel parameter selection to a convex optimization solution with multi-kernel functions. The set of multicore Gaussian kernel functions is as follows:

(7) $$\kappa =\left\{k=\sum_{\mu =1}^{m}\beta_{\mu }k_{\mu }\;|\;\sum_{\mu =1}^{m}\beta_{\mu }=1,\beta_{\mu }\geq 0\right\}$$

where the set of functions, κ, is defined as a convex combination of m Gaussian kernel functions with different kernel widths. ${\left\{\beta_{\mu }\right\}}_{u=1}^{m}$ is the convex optimization parameter set. In order to solve for the optimal value, the following constructor can be made:

(8) $$F\left(\beta_{\mu }\right)=\frac{\sum_{\mu =1}^{m}\beta_{\mu }k_{\mu }}{\frac{1}{m}\sum_{\mu =1}^{m}{\left(\beta_{\mu }k_{\mu }-\frac{1}{m}\sum_{\mu =1}^{m}\beta_{\mu }k_{\mu }\right)}^{2}}{\;k}_{\mu }\in \kappa$$

Then, when the constructor obtains the maximum value, a convex optimization problem exists:

(9) $$\underset{\beta_{\mu }}{max\;}F\left(\beta_{\mu }\right)$$

(10) $$s.t.\sum_{u=1}^{m}\beta_{\mu }=1\;\beta_{\mu }⩾0$$

Then, the min–max style of Equation (6) can be adapted to transfer the following probability distributions:

(11) $$\underset{\theta^{F}}{min}\underset{{\;k}_{\mu }\in \kappa }{max}{MMD}^{2}\left(F,X^{S},X^{T}\right)$$

### 3.3. The Training Method of WTDAN

#### 3.3.1. Loss Function

In order to measure the predictive performance of the model quantitatively, a loss function is often used to measure the discrepancy between the predicted results and the true results [27]. $L_{MMD}$ denotes the loss function of the MK-MMD distance between $X^{s}$ and $X^{T}$:

(12) $$L_{MMD}\left(\theta_{F}\right)={∥\frac{1}{n_{S}}\sum_{i=1}^{n_{S}}F\left(x_{i}^{S},\theta_{F}\right)-\frac{1}{n_{T}}\sum_{j=1}^{n_{T}}F\left(x_{j}^{T},\theta_{F}\right)∥}_{H}^{2}$$

where $x_{i}^{S}\;$ denotes the ith sample of $X^{S}$, and $x_{j}^{T}\;$ denotes the jth sample of $X^{T}$. $n_{S}$ is the number of samples in $X^{S}$, $n_{T}\;$ is the number of samples in $X^{T}$, and $\theta_{F}$ denotes the network parameters of the feature extractor.

Minimizing the distance between $X^{S}$ and $X^{T}$ allows us to minimize the loss function value:

(13) $$\underset{\theta_{F}}{min}L_{MMD}\left(\theta_{F}\right)$$

where $L_{C}$ denotes the loss function of the following label classifier:

(14) $$\begin{array}{ll} L_{C}\left(\theta_{F},\theta_{C}\right)= & -\frac{1}{n_{S}}\sum_{i=1}^{n_{S}}\sum_{j=1}^{n_{l}}p_{x_{i}\in j}\log\left(C\left(F\left(x_{i},\theta_{F}\right),\theta_{C}\right)\right)\\ & -\frac{1}{n_{T}}\sum_{i=1}^{n_{T}}\sum_{j=1}^{n_{l}}C\left(F\left(x_{i},\theta_{F}\right),\theta_{C}\right)\log\left(C\left(F\left(x_{i},\theta_{F}\right),\theta_{C}\right)\right) \end{array}$$

where $\theta_{C}$ denotes the network parameters of the label classifier. $n_{l}$ is the number of labels. $p_{x_{i}\in j}\;$ denotes that the probability $x_{i}$ belongs to category j.

$L_{D}$ denotes the loss function of the domain discriminator through the use of a binary cross-entropy loss function:

(15) $$L_{D}\left(\theta_{F},\theta_{D}\right)=-\frac{1}{n_{S}}\sum_{i=1}^{n_{S}}\log\left(D(F\left(x_{i}^{S},\theta_{F}\right),\theta_{D})\right)+\frac{1}{n_{T}}\sum_{j=1}^{n_{T}}\log(1-\left(D(F\left(x_{j}^{T},\theta_{F}\right),\theta_{D})\right))$$

where $\theta_{D}$ denotes the network parameters of the domain discriminator.

#### 3.3.2. Weight Factor Optimization

Unsupervised domain adversarial adaptive methods usually assign weight equally to every sample during the process of adaptation. When there are huge discrepancies between different samples, there is a high probability that there are several samples in the source domain that differ too much from the distribution to the target domain. The model may have a problem of negative transfer after training. In order to weight the different contributions to the model, we assigned different weights by measuring the discriminative difficulties of the domain discriminator. The harder the domain discriminator is to discriminate, the less weight is assigned to the source domain sample.$\;\omega_{i}^{s}\;$ denotes the weight value of the ith sample; this is expressed as follows:

(16) $$\omega_{i}^{s}=-\log(D(F(x_{i}^{S})))$$

Then, using the min–max normalization method, the normalized weight ${\tilde{\omega }}_{i}^{s}$ is:

(17) $${\tilde{\omega }}_{i}^{s}=\frac{\omega_{i}^{s}-\omega_{i,min}^{s}}{\omega_{i,max}^{s}-\omega_{i,min}^{s}}$$

where $\omega_{i,max}^{s}=max\left(\omega_{i}^{s}\right),\;\omega_{i,min}^{s}=min(\omega_{i}^{s})$. Let ${\tilde{\omega }}_{i}^{s}$ lead into Equation (11). Here, the loss function, ${\tilde{L}}_{D}$, is:

(18) $$\begin{array}{c} {\tilde{L}}_{D}\left(\theta_{F},\theta_{D}\right)=-\frac{1}{n_{S}}\sum_{i=1}^{n_{S}}{\tilde{\omega }}_{i}^{s}\log\left(D(F\left(x_{i}^{S},\theta_{F}\right),\theta_{D})\right)+\frac{1}{n_{T}}\sum_{j=1}^{n_{T}}\log(1-\left(D(F\left(x_{j}^{T},\theta_{F}\right),\theta_{D})\right)) \end{array}$$

Finally, the total loss function of the model is:

(19) $$L\left(\theta_{F},\theta_{C},\theta_{D}\right)=L_{C}\left(\theta_{F},\theta_{C}\right)-\mu {\tilde{L}}_{D}\left(\theta_{F},\theta_{D}\right)+\alpha L_{MMD}(\theta_{F})$$

where μ is a balancing parameter of the cross-domain error and α is an adjustment parameter.

#### 3.3.3. Training Strategy

There are two objectives in optimizing the parameters of the model: one is to identify the fault in target domain accurately, which involves minimizing the discrepancy in fault label classification; the other is to confuse the source domain with the target domain, which involves maximizing the discrepancy in domain classification:

(20) $$\left(\theta_{F}^{*},\theta_{C}^{*}\right)=arg\underset{\theta_{D},\theta_{C}}{min}L(\theta_{F},\theta_{C},\theta_{D}^{*})$$

(21) $$\left(\theta_{D}^{*}\right)=arg\underset{\theta_{D}}{max}L(\theta_{F}^{*},\theta_{C}^{*},\theta_{D})$$

where $\theta_{F}^{*},\theta_{C}^{*},\;and\;\theta_{D}^{*}$ represent the corresponding network parameters after the F, C, and D network optimizations, respectively.

Stochastic gradient descent (SGD) is used to update the parameters, as follows:

(22) $$\theta_{F}\leftarrow \theta_{F}-\varepsilon (\frac{\partial L_{C}}{\partial \theta_{F}}-\mu \frac{\partial {\tilde{L}}_{D}}{\partial \theta_{F}}+\alpha \frac{\partial L_{MMD}}{\partial \theta_{F}})$$

(23) $$\theta_{C}\leftarrow \theta_{C}-\varepsilon (\frac{\partial L_{C}}{\partial \theta_{C}}+\alpha \frac{\partial L_{MMD}}{\partial \theta_{C}})$$

(24) $$\theta_{D}\leftarrow \theta_{D}-\varepsilon \mu \frac{\partial {\tilde{L}}_{D}}{\partial \theta_{D}}$$

where ε is the learning rate. Due to the fact that gradient of the domain classification loss is in the opposite direction of the label classification loss gradient, the gradient reversal layer (GRL) reverses the gradient direction in the training process of backpropagation and achieves constant transmission in the forward-propagation process. This is expressed as follows:

(25) $$R_{\lambda }\left(x\right)=x$$

(26) $$\frac{{dR}_{\lambda }}{dx}=-\lambda I$$

where the parameter λ is not a fixed value and varies dynamically.

(27) $$\lambda_{p}=\frac{2}{1+\exp(-10\cdot p)}-1$$

where p denotes the relative value of the iteration process, i.e., the ratio of the current number of iterations to the total number of iterations. The growth of the parameters μ, and α obey the dynamic changes of $\lambda_{p}$.

**Figure 2:**
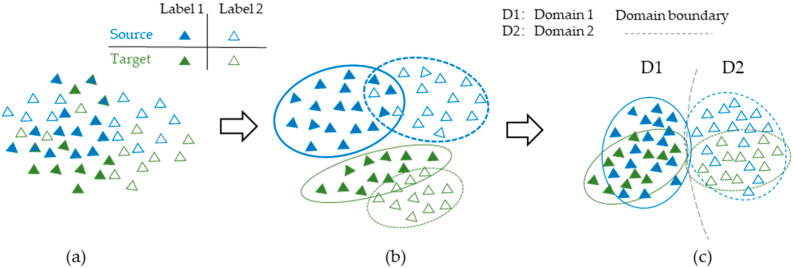
The training process of WTDAN. (**a**) The raw input; (**b**) the training process; (**c**) domain adaptation.

The training process of the method is shown in Figure 2. The first step aims to establish a nonlinear map from the source domain data sample, $X^{S}$, to the source labelling space; then, the model can obtain knowledge of the source domain, as shown in Figure 2b. The second task aims to build a cross-domain model that can eliminate domain distribution differences and extract domain-invariant features, as is shown in Figure 2c. Finally, the model achieves the ability to classify the unlabeled fault data from the target domain. 

## 4. Experiments and Results 

### 4.1. Descriptions of Datasets

**Figure 3:**
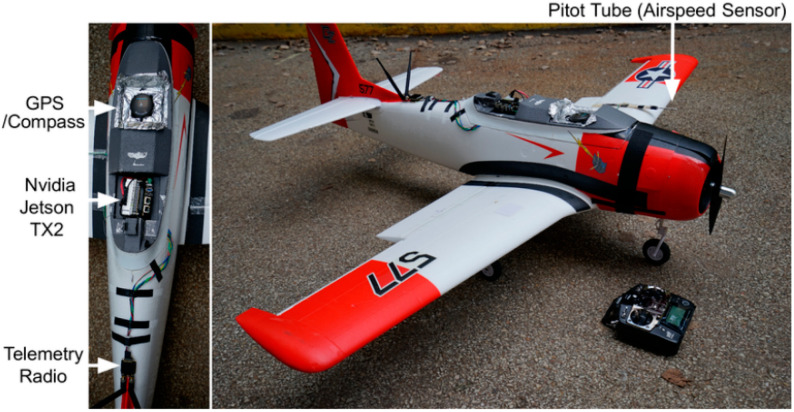
The flight test platform.

**Figure 4:**
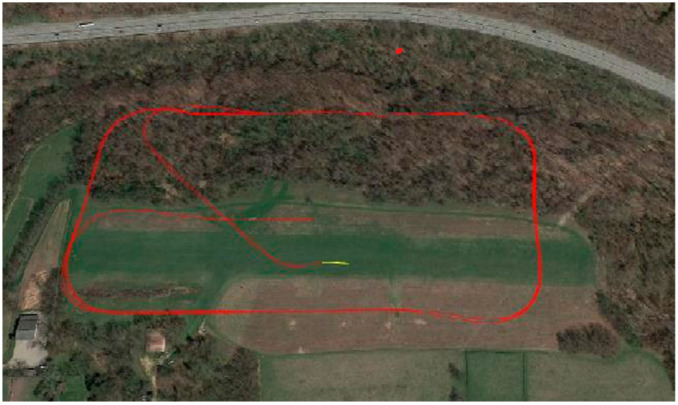
The flight path.

**Figure 5:**
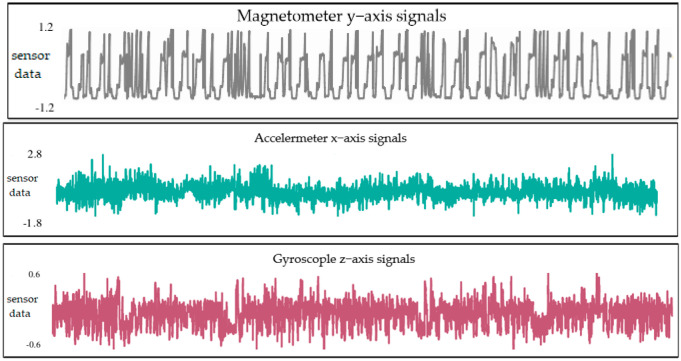
The data waveforms of sensor signals in the ALFA dataset.

It is difficult to obtain enough data to train a reliable fault-diagnosis model, since UAV sensor data from real scenarios are characterized by small samples with incomplete labels. Therefore, for this study, we selected the AirLab Failure and Anomaly (ALFA) dataset, an open database from Carnegie Mellon University [28], as the source domain dataset. This dataset records flight data from a small flight test platform, as shown in Figure 3. The flight test platform is equipped with a fixed-wing drone, a model called CarbonZ T-28. It has a 2 m wingspan, a central electric engine, a GPS module, a Pitot Tube airspeed sensor, and an Nvidia Jetson TX2; it uses Pixhawk as the flight-control system. The robot operating system collects signal data from the flight-control system and sends them to the ground station at regular intervals. The flight path taken during the test is shown in Figure 4. The dataset includes processed data for 47 autonomous flights with 23 sudden full-engine failure scenarios and 24 scenarios for other types of sudden control faults, with a total of 66 min of flight in normal conditions and 13 min of post-fault flight time. Additionally, it includes many hours of raw data of fully autonomous, autopilot-assisted, and manual flights, with tens of fault scenarios. The ground truth of the time and type of faults is provided in each scenario to enable the evaluation of new methods using the dataset. The ALFA dataset contains a variety of sensor raw data and fault data, in addition to the labels of fault diagnosis related to the sensors of this drone. Figure 5 illustrates the data waveforms of three sensor signals under normal conditions in the ALFA dataset.

The navigation subsystem, as a system for measuring the necessary flight parameters for UAVs, plays a key role in UAVs’ safe flight. Any errors obtained on the navigation subsystem can cause the system to send incorrect control commands. Considering the high frequency of this system, the sensor data on the navigation subsystem are selected from two types of datasets for training, validation, and testing.

In this experiment, four sensor-sensitive parameters on the navigation subsystem were selected from the ALFA dataset: airspeed, measured by an airspeed meter; x-axis linear acceleration, measured by an accelerometer; Y-axis magnetic field, measured by magnetometers; Z-axis angular velocity, measured by a gyroscope. Each type of sensor data contain four types of fault labels: constant (C), drift (D), instant (I), and bias (B). Each type of sensor data is cut into a length of 1024 datapoints, without overlap, to form a sample, with 500 samples comprising a dataset. Then, 80% of the samples in each type of dataset are randomly selected as the training part of the source domain dataset; the remaining 20% of the samples are used as the validation part of source domain dataset. 

The target domain data are obtained from real UAV flight tests. This dataset was collected by another flight test system. This flight test system mainly included a fixed-wing drone and a ground station. The drone model used was CG200, developed by CIOMP. The ground station used a portable computer; the model used was PT5214, made by INSUR. The ground station communicated with the drone by means of digital, graphic, and remote controls. The tests recorded flight data at various altitudes from 0 to 1000 m above sea level, with wind speeds between levels of 0 and 6, and outdoor temperatures between 10 °C and 20 °C, at different times and locations across Changchun, Jilin, China. 

Four sensor-sensitive parameters from the navigation subsystem were selected from the test dataset. The selected types of sensor data were the same as those selected in the above ALFA dataset. Each type of sensor data was cut into lengths of 1024 data points, without overlap, to form a sample, with 100 samples comprising a dataset. Of the samples in each type of dataset, 80% were selected randomly as the training part of the target domain dataset; the remaining 20% of samples were used as the validation part of target domain dataset. Then, another 100 samples were randomly selected to form the testing dataset. 

According to the fault-injection method proposed in Ref [29,30], we injected a certain percentage of faults into the sensor data of the validation dataset and test dataset, respectively, including constant, drift, instant, and bias faults. For the constant fault type, injected samples were generated by adding a constant offset value to the normal measured value of the sensor. We simulated the magnitude of the anomaly that was added to the base value at the onset of the anomaly using a random variable to capture various magnitudes in any given experiment. The magnitude of a given anomaly was sampled from a uniform distribution, $U\left(0,b\right),\;b\in \{0.5,1.5,3,20\}$. Various time lengths, d∈{1,5}, were set to the duration of the anomalous behavior. The drift anomaly type was simulated by adding a linearly increasing set of values to the base values of the sensors. We utilized a vector of linearly increasing values from 0 to c∈{2,4}, denoted by the function linspace(0,c). Various time lengths, d∈{10,20}, were set to the duration of the anomalous behavior. The instant fault type is often transient and unpredictable. Gaussian variables were randomly sampled from a standard normal distribution, $N\left(0,0.01\right)$, and the amplitude of the faults was controlled by the coefficients, $c\in \left\{25,100,500,1000\right\},$ in order to generate samples of different types from the instant fault samples. The bias anomaly type was simulated as a temporarily constant offset from the baseline sensor readings. We simulated the magnitude of the anomaly using a random variable to capture the various magnitudes; the magnitudes were sampled from a uniform distribution, $U\left(0,b\right),\;b\in \{0.5,1.5,3,20\}$. Various time lengths, d∈{3,5,10}, were set to the duration of the anomalous behavior; here, the sampled magnitude was added to all the true sensor readings during the specified duration to generate the anomalous readings. Both the labeled samples in the source domain and the unlabeled samples in the target domain were used for model training. The trained models were directly applied to the validation and test datasets. The dataset is shown in Table 1 below.

### 4.2. Environment and Settings 

The designed model used in the experiments, as mentioned above, contains three components: a feature extractor, a domain discriminator, and a label classifier. The feature extractor contains four convolutional blocks, a flatten layer, and a fully connected layer; each convolutional block is connected with a convolution layer, a maximum pooling layer, and an activation layer. The flatten layer is applied to transform the multidimensional data in the feature space into a one-dimensional data output. The domain discriminator contains three fully connected layers and a softmax function. The label classifier uses two fully connected layers and a softmax function. The network parameters are provided in Table 2.

In order to ensure that the experimental results are informative, all the following methods were conducted with the same parameter settings and the same structural settings. Each method was trained after 10 repetitive experiments, and each experiment was trained by 200 iterations. Using stochastic gradient descent (SGD) as the optimizer during the training process [31], we set the learning rates of the feature recognizer, the domain discriminator, and the label classifier to 0.001, 0.0001, and 0.0001, respectively. The SGD momentum was set to 0.85. μ,α were set to 1.2$\lambda_{p}$, 0.75$\lambda_{p}$, respectively. The source domain samples were pre-trained by 50 iterations before formal iterative training.

The processor Intel(R) Core(TM) i7-10870 CPU @ 2.20GHz with 64G of RAM was selected, running the Windows 10 operating system. The selected compilation environment was Pycharm; the proposed algorithm was implemented through the Pytorch framework, where Pytorch = 1.11.0 and python = 3.9.12.

### 4.3. Evaluation Indicators

Precision, recall, accuracy, and F1 score were selected for evaluating the performance of the model [32]: precision is the ratio of the actual true samples to the number of samples predicted to be true by the model; recall is the ratio of the actual true samples predicted to be true by the model; accuracy is the radio of the data predicted correctly by the model to the total data; F1 score is the harmonic mean of precision and recall. The formulas for these are as follows:

(28) $$Precision=\frac{TP}{TP+FP}$$

(29) $$Recall=\frac{TP}{TP+FN}$$

(30) $$Accuracy=\frac{TP+TN}{TP+FP+TN+FN}$$

(31) $$F1=\frac{2\times Precision\times Recall}{Precision+Recall}$$

TN, TP, FN, and FP represent the numbers of true negative, true positive, false negative, and false positive samples, respectively.

### 4.4. Model Training

**Figure 6:**
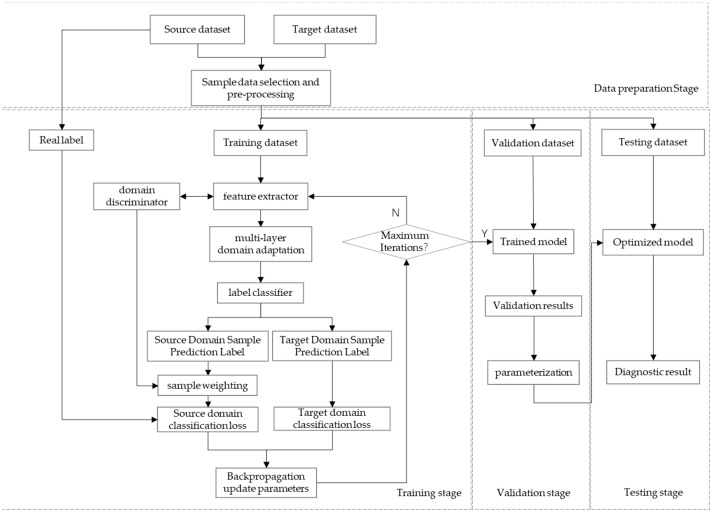
Flowchart of model training.

The training process of the proposed model consists of four stages: the data preparation stage, the training stage, the validation stage, and the testing stage. A flowchart of the stages is presented in Figure 6.

### 4.5. Test 1

In order to show the performance of model transfer learning, a total of six sets of migration trials, A→B, B→C, C→D, D→A, A→C, and B→D, were conducted. Using accuracy as a measure for modeling, each set of trials was conducted 10 times, obtaining the average of the total results.

**Figure 7:**
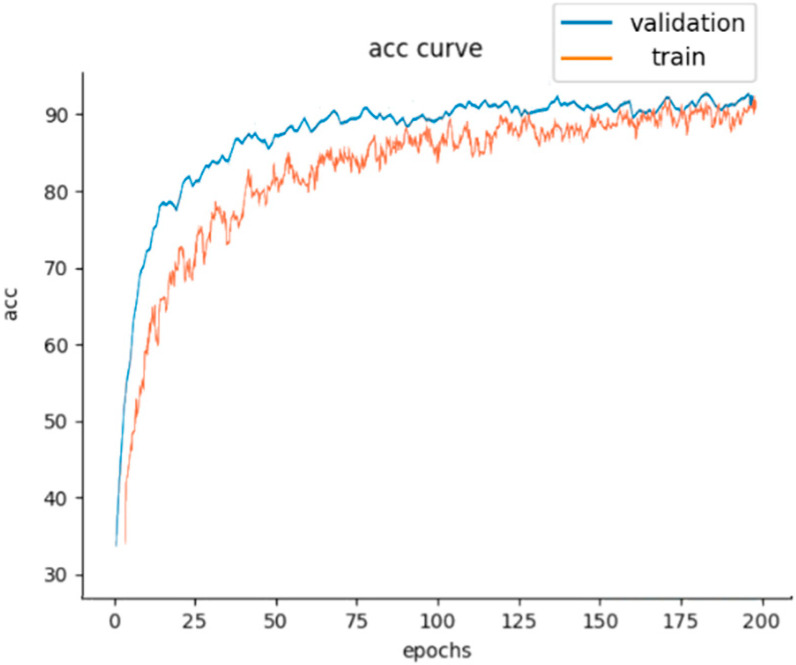
The training process of the acc curve.

**Figure 8:**
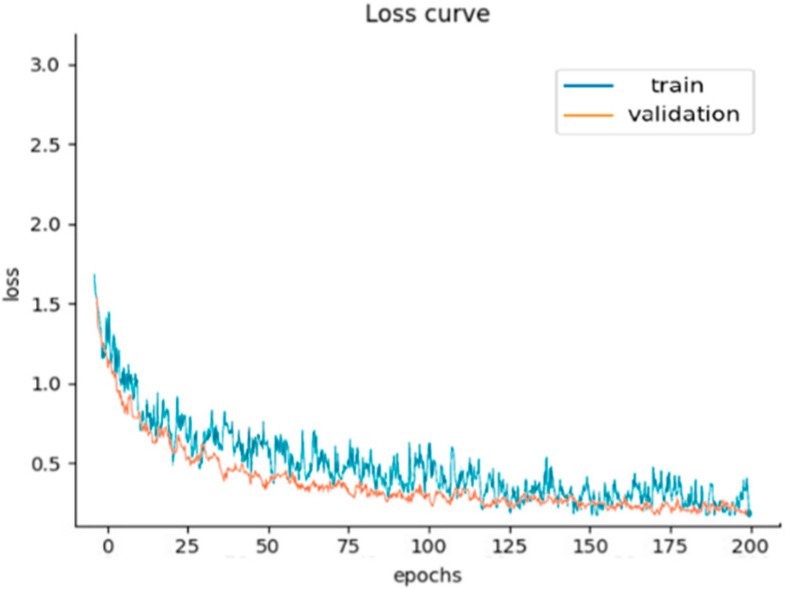
The training process of the loss curve.

As can be seen from Table 3, the method is able to maintain a cross-domain fault identification accuracy of more than 85% between different datasets; occasionally, it can exceed 90%. The network has a robust cross-domain identification of these four fault label types. Taking the A→B test as an example, the model accuracy and the loss value changes are shown in Figure 7 and Figure 8. As can be seen from Figure 7, in the training stage of the model, the label classifier’s training accuracy rises gradually with each iteration. The accuracy is close to 90% at about 120 iterations and tends to stabilize. The fluctuation amplitude of accuracy is obvious during the iteration process. Meanwhile, in the validation stage, the label classifier’s training accuracy is close to 90% at about 70 iterations and tends to stabilize. The fluctuation amplitude of accuracy is fine during the iteration process. This indicates that the model has the ability to predict the faulty labels on the extracted features after learning four types of signal samples gradually. As can be seen from Figure 8, the total loss value of the model decreases gradually with the increase in iterations. The parameters are continuously updated to decrease the difference between the source domain and target domain, eventually close to 0. This indicates that the discrepancy between the two trained domain datasets has been minimized, with the common features being mapped to the shared feature space, namely domain-invariant features.

### 4.6. Ablation Experiment

**Figure 9:**
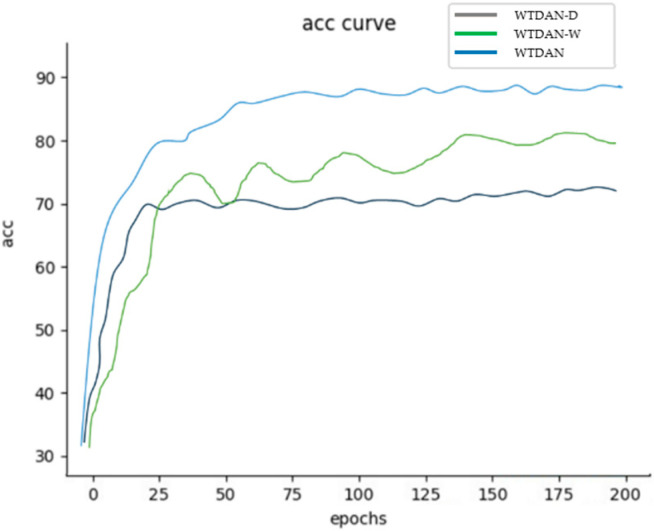
The confusion matrix of the test.

In order to assess the effectiveness of the models in the proposed method, we conducted ablation experiments for comparison. WTDAN−D denotes the method which removes the domain discriminator in WTDAN; WTDAN−W denotes the method which removes the weight factor in WTDAN. We test the two ablation methods with the same conditions as the A→B test in Test 1. The final accuracy results, as measured by WTDAN−D and WTDAN−W, are 70.58% and 84.41%, respectively. In order to highlight the main features of these accuracy curves, we conducted some smoothing of the experimental data curves. It can be seen from Figure 9 that the WTDAN−D fault recognition rate is at a low level; the model is unable to enrich small sample datasets or bridge the category imbalance differences. According to the WTDAN-W results, the training speed is slow and is not stable in the late stages of the training process; the model’s performance is turbulent due to the difficulty that arises in identifying negative samples and the underutilization of positive samples in the dataset.

### 4.7. Comparisons with Other Methods

In order to assess its effectiveness, six diagnostic methods were used for comparison to assess the transfer learning capability performance of the proposed method. One of the utilized comparative diagnostic methods was a convolutional neural network (CNN) [33]; this model is a pure deep learning method without transfer learning. The other comparative models included transfer component analysis (TCA) [34], a deep adaptation network (DAN) [35], and a dynamic adversarial adaptation network (DAAN) [36]. The test parameter settings were the same as those used in Test 1 (dataset source, number of iterations, and SGD parameter settings). The training set comprised 100 samples from each of the four source domain dataset types. The testing set comprised 100 samples selected randomly from the target domain dataset.

The experimental results in Table 4 show that the accuracy of the CNN method was lower than 50% in fault identification across the domains; this approach could not accurately detect the faults from the target domain samples. This finding indicates that traditional deep learning methods are unable to transfer enough feature information to the target domain to identify the feature of the target domain. The TCA method showed improved accuracy, exceeding 70%; this is double the accuracy achieved by the CNN. The DAN method achieved an accuracy of about 80%. This accuracy was further improved to 87% by the DAAN method. The proposed method achieved an accuracy of up to 90%; this is higher than those achieved by the comparative methods, indicating that the here-proposed method can effectively identify cross-domain faults in UAV sensors.

**Figure 10:**
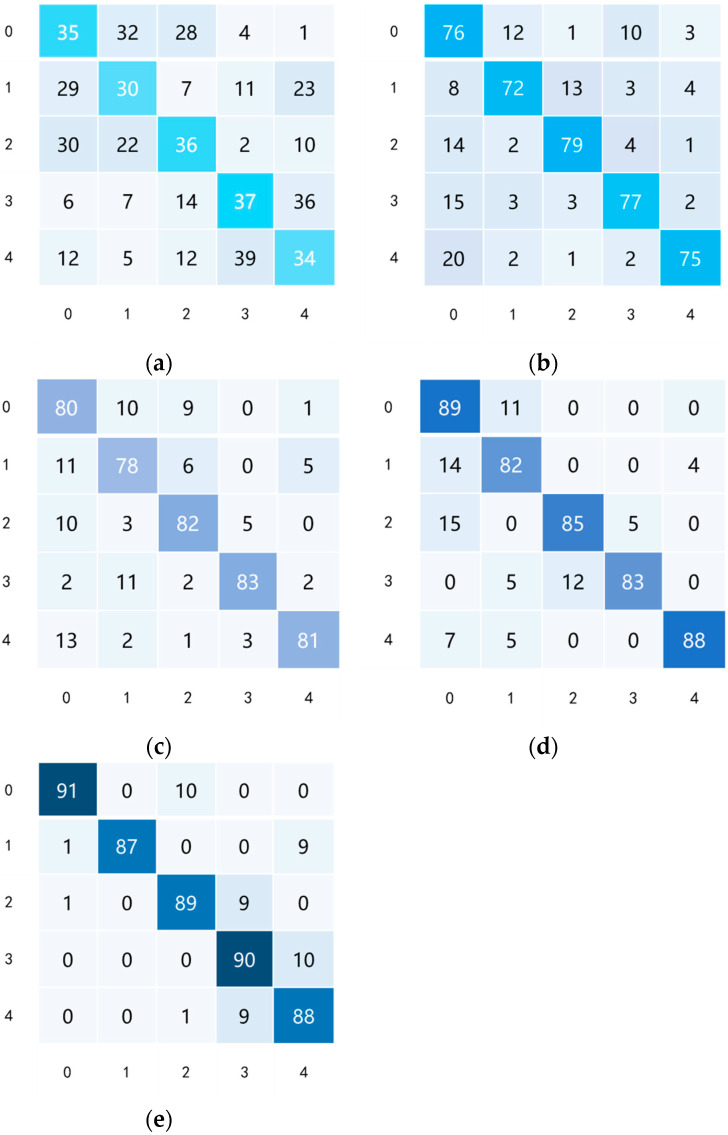
The confusion matrices of the test. (**a**) CNN method; (**b**) TCA method; (**c**) DAN method; (**d**) DAAN method; (**e**) WTDAN method.

To further show the performances of the different methods in fault diagnosis, the confusion matrices of the above tests were visualized [37]. There are 100 samples from each type of sensor, and a total of four fault label categories: No.0-4 correspond to the normal status and the four fault labels, as shown in Figure 10a–e. The horizontal coordinate is the predicted label, and the vertical coordinate is the true label. It can be seen from these confusion matrices that the enumerated methods have different performances in identifying the different sensor fault categories. Overall, the proposed method performs best for fault identification.

**Figure 11:**
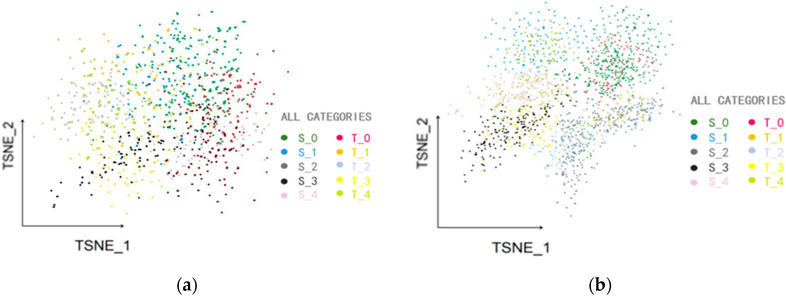

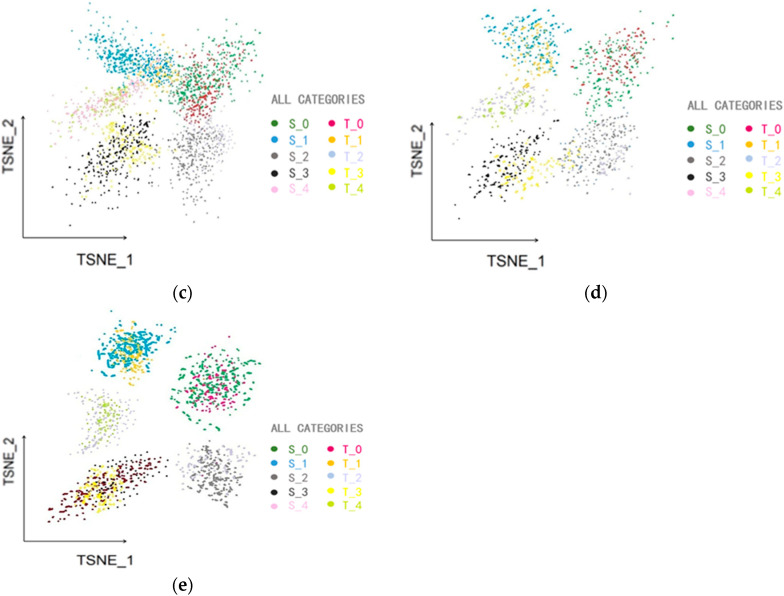
The t-SNE visualization of the tests: (**a**) CNN method; (**b**) TCA method; (**c**) DAN method; (**d**) DAAN method; (**e**) WTDAN method.

T-distributed stochastic neighbor embedding (t-SNE) can be used to visualize high-dimensional data by mapping samples in the original feature space to a two-dimensional space, clustering the same categories and separating different properties. In order to visually show the classification performances of these models, the test results for the five sets are visualized with t-SNE dimensional reduction [38], as shown in Figure 11a–e. The normal, constant, drift, instant, and bias fault labels are set to S_0, S_1, S_2, S_3, and S_4, respectively; labels from the target domain are set to T_0, T_1, T_2, T_3, and T_4, accordingly.

It can be seen from these t-SNE-visualized plots, although the feature points are somewhat concentrated in the two-dimensional space obtained by the CNN method, the clustering distribution is not focused, and different categories of features are mixed together; moreover, the separability of the different regions is unidentifiable. This indicates that, under the condition of having small data samples, the parameter optimization of the CNN-trained model is not enough to identify the fault features in the target domain. The different feature clusters extracted using the TCA method are more identifiable in the plots. However, the distribution of the feature points from the same samples is scattered over a wide area, and there is a significant overlap between the different categories. This indicates that the depth of the TCA network is low, and that it has a limited ability to extract high-dimensional features. So, the same feature cluster in the plot is not centralized. The clustering distribution of the feature points using the DAN method is more concentrated than it is using the TCA method. But the clustering distribution of the common feature points between the source domain and the target domain are partially aligned. This indicates that the method does not perform robustly in extracting the domain-invariant features, so the model is not accurate enough in some categories. The clustering effect of the feature points extracted by the DAAN method is further improved. The separability of the different clustering distributions is clear; most of the clustering distributions of common feature points between the source and target domains are aligned. However, there are still some overlapping edges and misalignments in the clustering distributions. This indicates that these methods can mostly adjust to domain adaptation, but the conditional probability distributions in domain adaptation require optimization. The clustering distribution of the features extracted by the proposed method is clear in two-dimensional space, the distribution of the same type of feature points is more centralized, and the separability of the different regions is good. And the clustering distributions of the common feature points are aligned well.

## 5. Discussion

In this paper, we have studied and validated a UAV sensor fault-diagnosis data-driven method using small-sample datasets; the proposed method has achieved better performance with flight data in comparison to other commonly used methods. There are many aspects of intelligent fault diagnosis that deserve to be explored due to the complexity of UAV structural components and the complexity of service work:

1. This case study concentrates on the fault diagnosis of UAV sensors; the next case study can be applied in other aspects of fault diagnosis, such as motors, gears, and bearings, to explore the application of transfer learning in the field of fault diagnosis.

2. In this paper, the input data were derived from a single-source domain; a future study will broaden this scope to include input data from multisource domains, continuously expanding the learning capabilities of the model. The future study should measure the relevance of the specified multisource domain to the target in some way; accordingly, we can ensure that attention is paid to those samples that are useful for the target domain and that we selectively filter out samples that have little relevance to the target domain. Here, we will adequately utilize our existing experience and knowledge from more relevant domains, ensuring that we train a better model.

3. In the future, we could explore the possibility of generating pseudo-labeled data in cases where the target domain contains a small amount of labeled data; then, we can set a threshold for retaining high-quality pseudo-labeled data, enriching the insufficiently labeled samples in the target domain.

4. In this paper, we mainly study the application of the proposed method on isomorphic types of data; meanwhile, other heterogeneous types of data, such as those for sound, video recordings, text, etc., are also correlated with fault features. Multimodal information fusion among heterogeneous feature space is a worthwhile aspect of research for transfer learning. Faults are often represented in more than one form, and we will explore the correlation between heterogeneous types of data features and faults in the future to enhance the robustness of the model for fault identification.

## 6. Conclusions

A novel weighted-transfer domain-adaption network is proposed in this paper. The model does not rely on the labeled data in the target domain; instead, it can obtain knowledge from the source domain to solve the problem that is faced in the target domain. The study examines the performance of the proposed WTDAN model through different transfer tests and compares its performance with those of other methods. The experimental results show that, under the condition of fewer target anomaly data samples, the proposed method presents higher classification accuracy on the target domain dataset and superior transferability on the cross-domain datasets than other compared methods. It can be concluded that, under a scarcity of labeled data in the target domain dataset, the proposed method has a good performance in online anomaly detection and fault diagnosis for UAVs’ electromagnetically sensitive flight data. The study provides a novel method for online anomaly detection and the prognostics and health management (PHM) of UAV fault diagnosis; in turn, this can improve the reliability, repairability, and safety of UAV systems.

## Figures and Tables

**Table 1:** The samples selected from ALFA dataset and target domain dataset.

Dataset	Sensor Type	Fault Categories	Labels	Samples
A	Airspeed meter	C/D/I/B	1/2/3/4	Train: 4 × 400 Test: 4 × 100
B	Accelerometer	C/D/I/B	1/2/3/4	Train: 4 × 400 Test: 4 × 100
C	Magnetometers	C/D/I/B	1/2/3/4	Train: 4 × 400 Test: 4 × 100
D	Gyroscope	C/D/I/B	1/2/3/4	Train: 4 × 400 Test: 4 × 100

**Table 2:** The network parameters.

Module	Block	Operator	Kernel/Stride /Channel	Output Size (Width, Depth)
Feature extractor	IN	Input data		1024, 1
	C1	Convolution	6 × 1/2/16	510, 16
	P1	Max pooling	1 × 1/3/16	170, 16
	A1	ReLu		170, 16
	C2	Convolution	3 × 1/1/32	168, 64
	P2	Max pooling	2 × 1/3/32	56, 64
	A2	ReLu		56, 64
	C3	Convolution	3 × 1/1/64	54, 64
	P3	Max pooling	1 × 1/3/64	18, 64
	A3	ReLu		18, 64
	C4	Convolution	6 × 1/1/128	12, 128
	P4	Max pooling	1/3/64/128	4, 128
	A4	ReLu		4, 128
	FL	Flatten	/	1, 512
	FC1	Dense	128	128
Domain discriminator	FC2-1	Fully connected	64	64
	FC2-2	Fully connected	32	32
	FC2-3	Fully connected	8	8
	O1	Softmax	2	2
Label predictor	FC3-1	Fully connected	32	32
	FC3-2	Fully connected	4	4
	O2	Softmax	4	4

**Table 3:** The performance of the model.

Test	Accuracy	Recall	F1 Score
A→B	90.58	90.97	90.69
B→C	87.41	88.12	87.95
C→D	89.08	90.1	89.95
D→A	88.7	89.4	89.05
A→C	90.2	91.1	90.64
B→D	89.74	90.28	90.94

**Table 4:** The performance of the proposed method compared with other methods.

Method	A→B	B→C	C→D	D→A	A→C	B→D	Average
CNN	35.82 ± 4.23	36.23 ± 4.18	33.45 ± 4.35	37.18 ± 4.03	34.97 ± 4.26	36.37 ± 4.17	35.67 ± 4.20
TCA	73.25 ± 1.35	77.12 ± 1.25	79.54 ± 1.17	72.75 ± 1.38	75.38 ± 1.28	78.12 ± 1.20	76.02 ± 1.27
DAN	81.85 ± 2.71	82.64 ± 2.49	79.87 ± 2.25	83.56 ± 2.78	80.05 ± 2.53	78.95 ± 2.29	81.15 ± 2.51
DAAN	80.17 ± 0.75	82.26 ± 0.68	85.18 ± 0.54	83.52 ± 0.61	86.08 ± 0.51	84.46 ± 0.58	87.61 ± 0.61
WTDAN	89.41 ± 0.41	90.24 ± 0.38	88.68 ± 0.47	91.16 ± 0.36	87.59 ± 0.52	88.27 ± 0.46	89.22 ± 0.43

## Data Availability

The original contributions presented in the study are included in the article; further inquiries can be directed to the corresponding author.
